# Recent Advances in Perioperative Analgesia in Thoracic Surgery: A Narrative Review

**DOI:** 10.3390/jcm14010038

**Published:** 2024-12-25

**Authors:** John Mitchell, Céline Couvreur, Patrice Forget

**Affiliations:** 1 Department of Anesthesiology, Catholic University of Louvain, University Hospital CHU UCL Namur, Mont-Godinne, 5530 Yvoir, Belgium; 2 Médecins Sans Frontières (MSF), Operational Centre Brussels (OCB), 1050 Brussels, Belgium; 3 Department of Emergency Medicine, Catholic University of Louvain, University Hospital CHU UCL Namur, Mont-Godinne, 5530 Yvoir, Belgium; celine.couvreur@chuuclnamur.uclouvain.be; 4 Aberdeen Centre for Arthritis and Musculoskeletal Health (Epidemiology Group), Institute of Applied Health Sciences, School of Medicine, Medical Sciences and Nutrition, Aberdeen AB24 3UE, UK; patrice.forget@abdn.ac.uk; 5 Anaesthesia Department, NHS Grampian, Aberdeen AB24 3UE, UK; 6 IMAGINE UR UM 103, Anesthesia Critical Care, Emergency and Pain Medicine Division, Nîmes University Hospital, Montpellier University, 30900 Nîmes, France; 7 Pain and Opioids after Surgery (PANDOS) European Society of Anaesthesiology and Intensive Care (ID ESAIC_RG_PAND) Research Group, 1000 Brussels, Belgium

**Keywords:** epidural analgesia, paravertebral block, thoracic surgery, erector spinae plane block, rib fracture

## Abstract

Thoracic surgery is associated with significant postoperative pain, which can hinder recovery and elevate morbidity risks. Traditionally, epidural anesthesia has been the cornerstone for pain management, but its drawbacks including technical challenges, side effects, and complications necessitate exploring alternative methods. This narrative review examined recent advances in perioperative analgesic strategies in thoracic surgery, focusing on regional anesthetic techniques like paravertebral blocks (PVBs), erector spinae plane blocks (ESPBs), intercostal blocks, and serratus anterior blocks. Each approach was evaluated for efficacy, safety, and impact on patient outcomes. PVB can provide effective unilateral analgesia with fewer systemic complications compared to epidurals. ESPB provides analgesia through a superficial, ultrasound-guided approach, minimizing risks and offering an alternative for various thoracic procedures. Intercostal blocks are effective but are limited by the need for multiple injections, increasing the complication risks. Serratus anterior blocks, targeting intercostal and thoracic nerves, show promise in managing lateral thoracic wall pain with a low complication rate. Advancements in surgical techniques including minimally invasive approaches further optimize pain control and recovery. A multimodal analgesic approach combining regional anesthesia and systemic therapies enhances outcomes by addressing somatic and visceral pain components. Despite the efficacy of epidural analgesia, alternative regional techniques offer comparable pain relief with fewer complications, suggesting their growing role in thoracic surgery. Collaborative efforts between surgical, anesthetic, and emergency teams are crucial for tailoring pain management strategies to individual patients, improving recovery and reducing long-term morbidity. Future research should continue exploring these methods to refine their application and broaden their accessibility.

## 1. Introduction

Thoracic surgery is widely regarded as one of the most severe postoperative pain procedures [1,2], and the related pain can greatly impair patient recovery and increase morbidity [3]. Traditionally, epidural anesthesia has been the cornerstone of perioperative pain management in thoracic surgery [4]. However, epidural anesthesia poses several challenges including technical difficulties, high failure rates, potential side effects, and complications [4,5,6]. This review aims to provide a comprehensive examination of alternatives to intraoperative and postoperative epidural anesthesia, focusing on their mechanisms, efficacy, benefits, and impact on patient outcomes. By considering various regional anesthesia techniques, describing them, comparing them, and analyzing their respective efficacies, this review aims to inform clinical practice and improve patient care in thoracic surgery. In the interests of clarity, in order to focus on recent advances and because the ultrasound tool is in full expansion in our operating rooms, the authors chose to focus on advances in locoregional anesthesia. Multimodal analgesia, although central to the analgesic strategy in thoracic surgery as in other surgical sectors, will therefore not be detailed here.

The authors have explored the literature, and based on their respective experience and expertise, discussed the selection and inclusion of papers according to their quality and impact.

## 2. Anatomical Considerations

Effective pain management in thoracic surgery requires a thorough understanding of thoracic anatomy. The thoracic cavity, surrounded by the rib cage, includes essential structures such as the heart, lungs, and major blood vessels. Key anatomical features relevant to regional anesthesia include the thoracic vertebrae, rib cage, intercostal spaces, spinal, vagus, and sympathetic nerves. The thoracic vertebrae provide structural support and are used as landmarks for various regional anesthesia techniques.

The thoracic cage, composed of the ribs, costal cartilages, and sternum, protects the thoracic organs and supports respiratory mechanics. The intercostal spaces, the areas between the ribs containing intercostal muscles, nerves, and vessels, are essential for various nerve blocks. The spinal nerves, emanating from the thoracic spinal cord, form the basis of many regional anesthesia techniques, with 12 pairs emerging from the vertebrae via the intervertebral foramina. They divide in the paravertebral space into an anterior and posterior branch. The anterior branch forms the extension of the intercostal nerve, which runs along the lower edge of the upper rib, itself giving rise to the lateral cutaneous branch (serratus anterior muscle) and the anterior cutaneous branch. The posterior branch crosses the costo-transverse ligament and the erector spinae muscles (iliocostalis, longissimus, and spinatus).

There are other important nerves for thoracic innervation and/or locoregional anesthesia at this level. The vagus nerve (or nerve X) carries afferences and efferences of the thoracic organs. Its role is vegetative regulation (digestion, heart rate, etc.) and sensorimotor control of the larynx, and therefore phonation. The phrenic nerves (C3–C5) are responsible for innervation of the diaphragm for the main respiratory movements, and for visceral afferences and efferences from the pericardium, diaphragmatic, visceral, and parietal pleura. The long thoracic nerve is formed by the anterior branches of spinal nerves C5–C7. This nerve is responsible for innervation of the superficial surface of the serratus anterior muscle. The thoracodorsal nerves are formed by the posterior bundles of the axillary plexus and are responsible for innervation of the latissimus dorsi muscle.

Finally, the sympathetic chain is located on either side of the spinal column. It modulates autonomic functions, playing a role in pain perception and autonomic regulation.

## 3. Thoracic Surgery and Pain Pathways

Thoracic surgeries including lobectomies, segmentectomies, and pneumonectomies, but also non-malignant disease such as empyema, trauma, and pneumothorax result in significant postoperative pain due to multiple factors. Extensive dissection and manipulation of tissues including muscles, ribs, and pleura contribute to postoperative discomfort. Rib dislocation and fractures during surgery can cause muscle and nerve injury. Intercostal nerve injury and pleural irritation during surgery by retractors, trocars, or drains (postoperatively) are a major source of pain. In addition, visceral pain results from the manipulation of thoracic organs.

Postoperative pain affects ventilatory mechanics, impairing deep breathing, coughing, and expectoration. It increases the risk of hypoxemia and hypercapnia and favors the development of atelectasis and pneumonia. It increases myocardial workload and the risk of arrhythmia or ischemia. Effective pain control is essential to facilitate respiratory function, allow for early mobilization, and prevent complications [1]. Respiratory complications of thoracic surgery include pulmonary atelectasis (5 to 10%), infectious pneumonitis (5–40% with 25% mortality), pulmonary embolism (0.5 to 5%; 25% mortality), acute respiratory failure (3%), hemothorax, pyothorax, mediastinal hematoma, bronchial fistula, and parenchymal breach. Cardiologic complications of thoracic surgery include atrial fibrillation (10 to 40%), acute pulmonary edema, and myocardial ischemia. Postoperative mortality is estimated at 2 to 6%, depending on the surgical procedure [7]. These cardiological and respiratory complications can lead to prolonged mechanical ventilation, reintubation, admission or readmission to intensive care, increased length of stay in intensive care and hospitalization, increased morbidity, and chronic post-thoracotomy pain. The incidence of chronic pain can reach 50% at 6 months and 20% even after 6 to 7 years [1].

Adequate pain relief is essential for rapid patient recovery, reducing the risk of chronic pain, and improving overall outcomes. Adequate pain relief therefore requires a multimodal approach that takes into account the somatic and visceral components of postoperative pain.

## 4. Evolution of Surgical Techniques

Thoracic surgical approaches have evolved from antero-lateral and postero-lateral thoracotomy to mini-thoracotomy, video-assisted thoracoscopy, and robot-assisted thoracoscopy. Thoracotomies can now be performed in a muscle-sparing manner, minimizing tissue disruption and improving postoperative recovery. Video-assisted thoracoscopy is also evolving in terms of trocar size (from 10 to 2–3 mm) and the number of trocars (from 3 to 1). Postoperative chest drainage can be performed less aggressively, using a single drain. The goal of all of these developments is to reduce surgical stress on tissues, postoperative pain, pleural drainage time, and length of stay [8].

## 5. Epidural Anesthesia

Epidural anesthesia is a local-regional anesthesia technique in which a catheter is inserted into the epidural space (the anatomical space surrounding the dura mater), allowing for prolonged diffusion of an active substance (usually a local anesthetic or an opioid). Still considered the gold standard in thoracic surgery, epidural anesthesia is widely used for analgesia in this kind of surgery, where the catheter is inserted into the epidural space around the T4–T5 or T5–T6 intervertebral spaces. There are two approaches (medial or para-medial) and a variety of techniques (loss of syringe resistance with saline and/or air, or the hanging drop technique). The goal is to relieve pain by blocking the somatic and visceral pain pathways at multiple levels and bilaterally. Insertion of an epidural catheter can be technically demanding, with a failure rate of up to 30%. The latter is explained by a complicated approach and possible catheter migration [5].

Contraindications to epidural use include sepsis or infection at the puncture site, coagulopathy or use of all anticoagulant medications, pre-existing neurological diseases, and variations in spinal anatomy [9]. Common side effects of thoracic epidural include hypotension (7% of cases) [4], nausea and vomiting, urinary retention, pruritus, and motor block, all of which can interfere with early patient mobilization [10]. Major complications have an incidence of 1/6000 to 1/1000 and include spinal cord hematoma, spinal cord abscess, and spinal cord puncture [6].

Given these limitations and the advances in surgical (minimally invasive techniques) and anesthetic technology (advent of ultrasound-guided locoregional anesthesia), and even if the epidural remains the gold standard and can therefore be offered in almost all thoracic surgical indications (except where there are contraindications to the epidural), alternative regional anesthesia techniques have been developed in an attempt to provide effective analgesia with fewer complications.

## 6. Alternatives to Epidural Anesthesia: Regional Anesthesia Approaches

### 6.1. Paravertebral Block (PVB)

The paravertebral block (PVB), described in 1905 by Selheim and Lawen, consists of injecting local anesthetics into the paravertebral space, close to the spinal nerve roots, just after they exit the spinal canal [11]. The paravertebral space (PVS) is a triangular-shaped anatomical space on a transverse anatomical section, whose anterior wall consists of the parietal pleura. The bodies of the vertebrae, the intervertebral discs, and the intervertebral foramen represent the medial wall. The posterior wall consists of the superior costo-transverse ligaments (SCTLs) and the costo-transverse joints (costo-transverse processes). The PVS extends laterally into the intercostal space. The PVS communicates with the inferior and superior spaces and terminates at L1 (at the origin of the psoas muscle). Its cephalic termination is more imprecise. The PVS contains the spinal (intercostal) nerve, the dorsal and communicating branches, and the sympathetic chain adjacent to the thoracic vertebrae [12].

**Figure 1:**
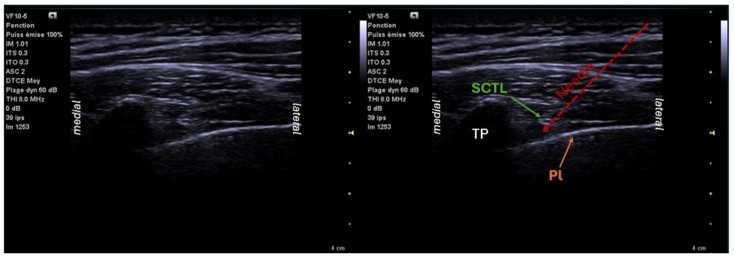
Ultrasound location for paravertebral block: transversal view T4–T5. TP: transverse processes; Pl: pleura; SCTL: superior costo-transverse ligament.

Historically, the PVB was quickly overtaken by epidurals. In 1979, Eason and Wyatt re-evaluated the technique by including a catheter, allowing continuous perfusion [13]. The technique of PVB placement involves a posterior puncture 2 to 3 cm from the midline lateral to the line of the spinous processes, through the paravertebral muscles and the superior costo-transverse ligament (SCTL), using a Tuohy needle and the loss of resistance technique. An advance is then made perpendicular to the plane until bony contact is established with the transverse process (15 to 40 mm). The needle is then mobilized in a cephalad direction and advanced until it crosses the LCTS and loses its resistance (24 to 56 mm) [14]. After aspiration to ensure that the catheter is not in a vessel, the LA can be injected slowly and fractionally. A catheter can also be inserted. PVBs can be performed under ultrasound guidance. In this case, the square-shaped transverse process, which produces a shadow cone, and the hyperechoic and mobile pleura are the main landmarks (Figure 1). The probe is positioned so as to be able to visualize a cross-section of the epidural space at the desired metamere; we could distinguish the median transverse process and its shadow cone, the hyperechoic and mobile pleura in the background of the image, the superior costotransverse ligament connecting the rib and the transverse apophysis, and running in front of the pleura. The needle is introduced in the plane of the ultrasound probe, positioned transversely toward the paravertebral space, most often starting from the outside and directing the needle toward the median axis. Injection into the plane after an aspiration test causes pleural collapse, which is then pushed back toward the back of the image [13,15,16]. Finally, the PVB can be performed by the surgeon under direct vision via the intercostal space [17].

The PVB provides effective unilateral analgesia over multiple thoracic metameres in a single injection by cephalad and caudal diffusion, the PVS being an unenclosed space. Its effect is accompanied by anesthesia of the sympathetic ganglion chain, producing sympathetic block and vasodilation [18]. Its unilateral action reduces the risk of hypotension and motor block compared with epidural anesthesia. PVB is considered simple (failure rate: 6 to 10%) but has a long learning curve. Complications include intravascular puncture, hypotension, hematoma, pneumothorax, pleural, perimedullary, or spinal puncture [19]. As the PVB remains an incompressible space like the epidural space, abnormalities of hemostasis contraindicate its use. Other contraindications to PVB are infection at the puncture site and pleurectomy. Postoperative management of continuous paravertebral block is possible in a standard hospital unit (versus the recovery room) [20].

In a 2006 meta-analysis of 10 studies and 520 patients comparing PVB and thoracic epidural for thoracotomy, Davies et al. showed no significant difference in pain scores. The rate of pulmonary complications, urinary retention, PONV, and hypotension was significantly lower in patients receiving analgesia with PVB. The failure rate was lower in the PVB group. The authors concluded that PVB can be recommended for thoracic surgery [21]. In a 2008 systematic review of 74 studies, Joshi et al. concluded that a paravertebral block is an effective alternative to thoracic epidural with local anesthesia alone, but that further studies were needed to determine whether PVB is equivalent to a thoracic epidural with combined local anesthesia and opioids in terms of pain relief and morbidity [22]. Another meta-analysis by Yeung from the Cochrane Library in 2016 including 14 studies and 698 patients concluded that thoracic epidural and PVB were equally effective in controlling pain during thoracotomy surgery, with a significantly lower incidence of minor complications (hypotension, ileus, sedation, PONV, pruritus, urinary retention) in the PVB group. However, she qualified this by indicating a lack of evidence for other outcomes such as 30-day mortality and major complications (cardiovascular, pulmonary, neurological, ICU readmission, repeat surgery) [17].

### 6.2. Erector Spinae Plane Block (ESPB)

The erector spinae muscles include the iliocostalis muscle in the lateral part of the paravertebral groove, the longissimus muscle in the middle part of the paravertebral groove, and the spinatus muscle in the medial part of the paravertebral groove.

The erector spinae block (ESPB), first described in 2016 by Ferrero et al., involves the injection of local anesthetics in the plane between the erector spinae muscles and the transverse processes of the vertebrae, where, as a reminder, the dorsal branch of the spinal nerve extends [23]. Its effect is analgesia from the back to the midline of the axilla [24]. Due to its superficial approach and always performed under ultrasound, it is technically easy to perform with a favorable safety profile and minimal side effects [23], the injection of local anesthetics being performed at a distance from noble elements such as the neuraxis and the lung, at the level of the paraspinal space. This paraspinal space communicates with the paravertebral space via the costovertebral foramen, the interstices between the muscles and the costo-transverse ligaments. The nerve targeted during this block is therefore the dorsal branch of the spinal nerve, but also and by extension the ventral branch, the intercostal, and paravertebral spaces. This technique provides somatic and visceral analgesia by spreading to the paravertebral, epidural, and intercostal spaces, resulting in analgesia from the posterior part of the chest wall to the middle of the axillary line, with a possible anterior extension [15].

**Figure 2:**
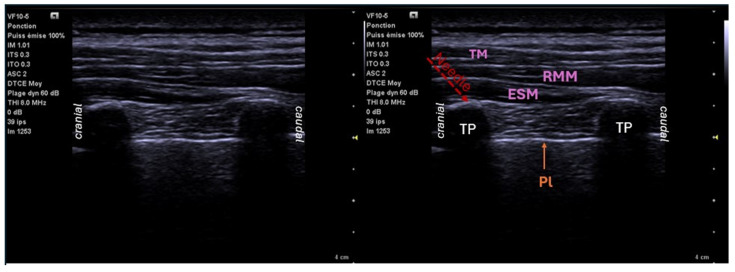
Ultrasound location for erector spinae plane block: sagittal view T4–T5. TP: transverse processes; Pl: pleura; ESM: erector spinae muscle; RMM: rhomboid major muscle; TM: trapezius muscle.

The technique for thoracic surgery involves placing the needle under ultrasound visualization between the posterior edge of the distal end of the square-shaped transverse process of the vertebra and the fascia of the erector spinae muscles at T4–T5. The only important ultrasound landmark is the transverse process, knowing that above this transverse process are always the erector spinae muscles between C5 and L5 (Figure 2). It is important to really try to detach the space by taking advantage of the transverse process and not to inject into the muscle mass. The placement of a catheter is possible. Due to the anatomy and extension of the muscles along the spine, the local anesthetic diffuses in a cranio-caudal plane, acting as a reservoir on several dermatomes, but also toward the PV space, the costo-vertebral foramen, the epidural space, and the intercostal space [25]. It is crucial to inject a sufficient volume (3.4 mL/metamer (9 metameres = 30 mL)) as it is this significant diffusion that ensures the success of the block [26].

The success rate is 78% [27]. The overall complication rate is estimated at no more than 2 cases per 10,000 patients [28], and these include motor blocks [29], pneumothorax [30], intravascular injection, and systemic toxicity [31]. This block can be complicated by a hematoma, but this is of little importance because the injection space is compressible and far from the neuraxis [32]. Similarly, the risk profile is considered low as the injection is carried out far from the pleura, spinal cord, or vessels.

A prospective randomized study, published by van den Brock et al. in 2024, compared ESPB to thoracic epidural during VATS surgery in 90 patients. The study concluded that ESPB was non-inferior to epidural in terms of quality of recovery (QoR-15), pain score, length of hospital stay, morphine consumption, occurrence of PONV or pruritus, speed of mobilization, and need for urinary catheterization [33]. A retrospective study by Durey et al. in 2023 including 107 patients undergoing thoracic surgery by VATS or RATS compared ESPB to paravertebral block and showed no difference in terms of morphine consumption and complications at H24 postoperatively. The authors concluded that ESPB is an acceptable and safe alternative to PVB [34]. Additionally, in a prospective, double-blind, randomized, controlled study including 80 patients undergoing VATS and published in 2023, Moorthy et al. compared the effectiveness of anesthesiologist-administered ultrasound-guided ESPB with surgeon-administered video-assisted PVB. The authors concluded that compared with surgeon-placed PVB, the ESP catheter improved the overall quality of the recovery scores (QoR-15 scores) at 24 h and 48 h, but without differences in pain or opioid consumption [35].

It has also been shown to be effective for the management of acute and chronic pain in thoracic surgery [23].

### 6.3. Intercostal Block

**Figure 3:**
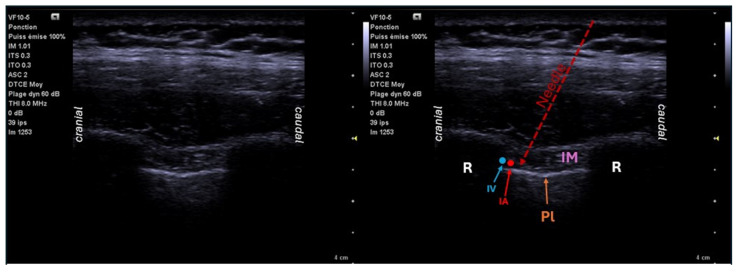
Ultrasound location for intercostal block: sagittal view T4–T5. R: rib; Pl: pleura; IM: intercostal muscle; IV: intercostal vein; IA: intercostal artery.

Described in 1907 by Braun et al., the intercostal block (ICB) is based on the principle of injecting close to the intercostal nerve. It can be performed percutaneously by the anesthesiologist (with or without ultrasound) or by the surgeon under direct visual control. The success rate is 70 to 97%, depending on the technique used (without versus with ultrasound) [36]. This block has two major disadvantages. It must be performed in stages over 5 to 6 levels (LA volume: 3–5 mL per level) [3] because it only extends laterally. The need to repeat the puncture increases the risk of intravascular or pleural puncture. The injection is also close to many vessels, which increases the risk of local anesthetic intoxication through rapid resorption. This rapid resorption reduces the duration of action of the ICB. It is possible to place a catheter in the intercostal space when performing this block, but the failure rate is 20%. Complications of ICB include pneumothorax (0.073 to 19%; 1.4%/injection), rarely compressive, nerve injury, mainly present if the block is performed without ultrasound, vascular puncture, hemothorax, LA toxicity, and rare cases of infection at the puncture site [36]. The contribution of ultrasound reduces these risks by visualizing the pleura and the neurovascular bundle (Figure 3). The surgeon’s ability to perform the block reduces these risks and speeds up the procedure [3].

A 2005 randomized trial involving 91 patients compared surgeon-initiated ICB with thoracic epidural in thoracotomy surgery. Results showed no difference in pain scores. The number of days requiring urinary catheterization was greater in the epidural group. There was no difference in terms of complications such as arrhythmia, pneumonia, need for transfusion, and antibiotic use. There was no difference in terms of post-operative respiratory function, length of stay in the recovery room, or length of hospital stay [37].

A 2021 systematic review and meta-analysis including 69 articles and 5184 patients compared ICB to systemic analgesia, epidural, and PVB. In its primary outcomes, this study showed lower pain during the 24 h after surgery in the ICB group than in the systemic group, a clinically non-inferior efficacy of intercostal block compared to epidural and PVB, and a morphine-sparing effect in the ICB group. However, this effect was greater for the epidural or PVB groups. In the secondary endpoints, PONV was less common in the PVB and epidural groups than in the ICB group but more common in the ICB group than in the systemic group. The risk of cardiovascular complications was similar for the ICB group and the systemic group, but was less in the ICB group compared with the epidural group. The incidence of hypotension was lower in the ICB group than in the epidural group. Pulmonary complications were as common in the ICB group as in the epidural or PVB groups but remained less significant in the ICB group than in the systemic analgesia group. The 30-day mortality was identical in the ICB group and in the epidural group. The incidence of neurological complications, pruritus, catheter or puncture site infections, hematomas, and urinary retention was identical in the ICB group compared to the epidural group. This article concluded that the use of the ICB was especially indicated in the case of contraindication to the epidural and PVB [38].

A systematic review and meta-analysis from 2023 of 9 studies including 498 patients compared the ICB to the thoracic epidural. This study showed no difference in terms of pain score, incidence of PONV, morphine consumption, or length of hospital stay. Therefore, the authors concluded that IC was as effective as thoracic epidural for post-thoracotomy pain [39].

### 6.4. Serratus Block

**Figure 4:**
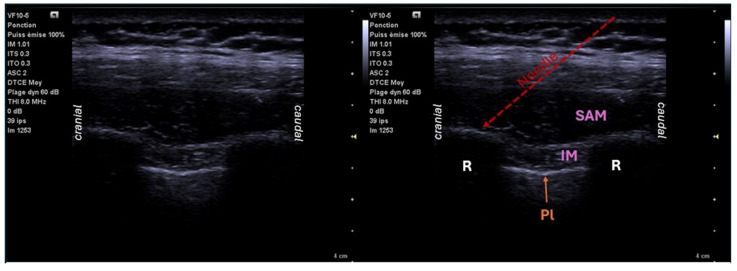
Ultrasound location for serratus block: sagittal view T4–T5. R: rib; Pl: pleura; IM: intercostal muscle; SAM: serratus anterior muscle.

The serratus anterior muscle is rectangular in shape and extends from the lateral aspect of the first nine ribs to the medial aspect of the scapula. It is crossed at the costal angle by the lateral cutaneous nerves (collaterals of the intercostal nerves) innervating the lateral thoracic flank and the long thoracic and thoracodorsal nerves that descend on the surface of the muscle. First described in 2013 by Blanco, the serratus plane block (SB) is an ultrasound-guided regional anesthesia technique that can achieve complete paresthesia of the hemithorax and provide analgesia following surgery on the thoracic wall [40]. The serratus anterior block technique aims to block the innervation of the lateral branch of the IC nerve in its peripheral part under ultrasound guidance at the level of the fifth IC space on the mid-axillary line. The injection is performed under the serratus muscle or above the serratus muscle (between the serratus muscle and the latissimus dorsi muscle). It blocks the long thoracic nerve and the lateral branches of the intercostal nerves. This injection is superficial and considered simple. There are variations of the serratus anterior block. The superficial serratus block is performed on the mid-axillary line after ultrasound identification of the serratus anterior and latissimus dorsi muscles. The injection is performed in the fascia between the two muscles. The deep serratus block requires the same needle placement, but the injection is performed under the serratus anterior muscle, in contact with the rib, with a possible effect on the external intercostal muscles (Figure 4). A modified version described in 2016 [41] shows an approach on the posterior axillary line at the level of the sixth rib, an injection between the latissimus dorsi muscle and the serratus anterior muscle, and a more posterior approach to reach the thoracodorsal nerve. The diffusion of LA during SB allows for analgesia of the hemithorax from the second to the ninth intercostal dermatome. Content of the lesion is limited to the lateral cutaneous branches, and deep visceral pain is not relieved [42]. The volume of local anesthesia to be injected is 20 to 40 mL. Catheterization is possible. SB is considered safe, effective, and easy to perform, and is associated with a low risk of side effects [40]. The risks associated with performing SB are low because it is performed far from the pleura, vessels, or major nerves. No complications were described in a 2020 meta-analysis of 875 cases [43]. The success rate of SB is 86.4% [27].

A 2017 prospective randomized study including 40 patients compared serratus anterior block to thoracic epidural during thoracotomy surgery and showed no significant difference in terms of pain score during the first 24 postoperative hours or in terms of morphine consumption. The authors concluded that SB represents a safe and effective alternative epidural in the post-thoracotomy setting [44]. In a 2023 systemic review and meta-analysis of 12 studies and 837 patients, the serratus anterior block was compared to systemic analgesia during video-assisted thoracoscopy. This article showed a significant decrease in pain score during the first 24 postoperative hours, opioid consumption during the first 24 postoperative hours, and PONV by reduction in opioid consumption. There was no difference in length of hospital stay. The authors concluded that the addition of a serratus anterior block decreased the pain scores, opioid consumption, and the incidence of PONV during VATS [45]. A 2023 systematic review and meta-analysis of 6 studies and 384 patients compared the serratus anterior block to thoracic epidural. This study showed comparable VAS pain scores between the serratus anterior block and thoracic epidural. In this study, the incidence of hypotension was lower in the serratus group and no difference was demonstrated in the incidence of PONV. The authors concluded that the serratus anterior block may be an alternative to epidural in thoracic surgery [46].

The main articles for each analgesic technique have been listed in Table 1 in order to quickly access the key studies supporting the use of each technique.

## 7. A Word on Parasternal Block

Although the parasternal block is mainly used in cardiac surgery, it can also be used in thoracic surgery. The sternal region is innervated by the anterior branches of the intercostal nerves, which arise from the anterior branches of the spinal nerves from T1 to T11. The parasternal block targets these branches by injecting local anesthetic between the pectoralis major and the intercostal muscles, proximal to the sternum. To obtain good analgesic coverage, the block must be performed bilaterally with diffusion from the second to the sixth intercostal space [47]. Mediastinal tumors are rare pathologies. Anterior mediastinal tumors cause serious and often fatal complications related to compression of the airways and vascular structures. General anesthesia can aggravate these complications. The rate of these can then reach 7 to 20%. The replacement of general anesthesia by locoregional techniques makes it possible to limit these complications, both during the induction phase of anesthesia and following the use of certain anesthetic drugs. Thoracic epidural anesthesia, intercostal nerve blocks, and paravertebral blocks are the most common locoregional techniques, used alone or in association with other techniques at different levels of consciousness [48], which have been previously mentioned. However, although it is mainly used in cardiac surgery, the parasternal block appears as an additional alternative solution. Although it has shown limitations for analgesia of the epigastric region in cardiac surgery [49], it has also reduced the need for opioids to ensure hemodynamic stability in this kind of surgery [50]. Finally, a randomized, double-blind, placebo-controlled trial by Chen et al. in 2021 demonstrated that the parasternal block was a good option for the resection of mediastinal masses by median sternotomy by reducing postoperative pain and the need for adjuvants [51].

## 8. A Word on Multimodal Analgesia

Multimodal analgesia combines opioids with other non-opioid antihyperalgesic analgesics as well as locoregional analgesia techniques. Its main objective is to seek a synergistic analgesic effect via opioids, paracetamol, non-steroidal anti-inflammatory drugs, N-methyl-D-aspartate (NMDA) antagonists, alpha-2 agonists, gabapentinoids, and locoregional anesthesia. All of these medications, except for locoregional anesthesia, are the basis of systemic analgesia and can be used in the management of pain in thoracic surgery [3,52].

## 9. Pain Management After Rib Fracture

Whether treated or not by thoracic surgery, rib fractures are also a major cause of pain. Rib fractures are the most frequent type of thoracic injury. A total of 65% of the mortality associated with these fractures is not directly related to the trauma but is attributed to secondary pulmonary complications (infection induced by ineffective cough, failure of respiratory mechanics) [53]. Better pain relief is essential to avoid these complications.

Multimodal analgesia improves pain scores and pulmonary ventilation [54], but intravenous analgesia has many side effects (sedation, decreased cough, respiratory depression, opioid dependence) [55]. Several locoregional anesthesia techniques are used to relieve pain associated with rib fractures in emergency departments, although epidural analgesia has been the most extensively studied. In a systematic review in 2024, Hammal et al. concluded that the pain scores were significantly lower with epidural analgesia than with other modalities [56].

Epidural anesthesia was compared to paravertebral block by Mohta et al. in 2009 [57]. This study showed a similar efficacy between the two techniques in terms of pain scores, ventilatory parameters (respiratory frequency, FEV1, PaO_2_/FiO_2_), pulmonary complication rates, and length of hospital stay. However, paravertebral block showed superiority in all of these areas compared to intravenous analgesia alone [58].

Fascial blocks have also been studied for pain associated with rib fractures. Serratus anterior blocks were superior to standard treatments (without locoregional anesthesia) in terms of pain score and use of tramadol [59] and opioids [60]. Lunden et al. showed in 2023 that epidural analgesia was superior to an ultrasound serratus anterior block in the management of pain in the first twelve hours after rib fractures [61]. In contrast, the SABRE study published in 2024 clearly demonstrated the superiority of the serratus anterior block over standard care in terms of pain score and morphine use [60]. To date, erector spinae blocks have not been directly compared to epidural analgesia. The study by Ramesh et al. in 2024 comparing it to a placebo failed to demonstrate its superiority [62].

In a 2011 study, Hashemzadeh showed that epidural analgesia was superior to intercostal block in terms of ventilatory parameters, pain scores, and length of hospital stay [63]. In addition, intercostal block requires multiple injections in cases of multiple rib fractures, and therefore increases the risk of complications (pneumothorax and vascular injuries) [64].

Although less frequently used, interpleural block (using a catheter inserted directly next to the chest drain in cases of hemothorax associated with rib fractures) was compared to epidural block in 1994 by Shinohara, demonstrating a similar efficacy between the two techniques [65]. The risk of infection and the need to remove the catheter during drain mobilization are two of the reasons why this technique is not widely used.

However, as in thoracic surgery, coagulation problems (particularly of medical origin) remain a barrier to the use of epidural analgesia. More peripheral blocks have the advantage of not being contraindicated in this case. Several studies have also shown a higher rate of arterial hypotension under epidural analgesia than with other modalities [57,65,66].

Finally, emergency physicians are often not the clinicians best trained in locoregional anesthesia. Konrad showed that 90 attempts at epidural insertion are necessary to achieve an 80% success rate [67]. Collaboration between the emergency and anesthesia departments is therefore essential for these trauma patients.

## 10. Conclusions

Thoracic surgery is painful despite advances in this area. There is a wide arsenal of medications available but all have side effects, risks of complications, and a high failure rate. The combination of locoregional anesthesia with systemic analgesia is now widely recommended in the multimodal management of pain in thoracic surgery. Epidural has long been the gold standard technique for locoregional anesthesia, however, it has side effects and can be a difficult technique to implement. The advent of ultrasound has allowed for new blocks. Many alternatives to thoracic epidural exist for the management of pain in thoracic surgery. Each has its own indications, contraindications, and potential complications. The choice of an alternative locoregional technique for the management of the patient must be made by taking into account the ability of the practitioner, patient-related considerations, surgical considerations, and the habits of the multidisciplinary team caring for the patient intra- and postoperatively. Rib fractures are the most common type of thoracic injury. The same regional anesthesia techniques as those used for thoracic surgery can be used to treat pain. Collaboration between the emergency and anesthesia departments is essential for these trauma patients.

## Figures and Tables

**Table 1:** Main articles comparing locoregional analgesia techniques to epidural for thoracic surgery (selected by the authors according to their expertise).

Techniques	Compared to	Authors	Year	Journal	Type
Paravertebral block	Epidural	Davies et al. [21]	2006	British Journal of Anesthesia	Systematic review and meta-analysis
	Epidural	Joshi et al. [22]	2008	Anesthesia Analgesia	Systematic Review
	Epidural	Yeung et al. [17]	2016	Cochrane Database	Systematic Review
Erector spinae plane block	Epidural	Van den Broek et al. [33]	2024	Regional Anesthesia & Pain Medicine	Prospective randomised trial
	Paravertebral	Durey B. Et al. [34]	2023	Cancers	Retrospective trial
	Paravertebral	Moorthy et al. [35]	2023	British Journal of Anesthesia	Prospective randomised trial
Intercostal block	Epidural	Luketich et al. [37]	2005	The Annals of Thoracic Surgery	Randomised trial
	Systemic, epidural, paravertebral	Guerra-Londono et al. [38]	2021	Journal of the American Medical Association	Systematic review and meta-analysis
	Epidural	Zhou et al. [39]	2023	Pain Physician	Systematic review and meta-analysis
Serratus block	Epidural	Khalil et al. [44]	2017	Journal of Cardiothoracic and Vascular Anesthesia	Prospective randomised trial
	Systemic	Li et al. [45]	2023	BMC Anesthesiology	Systematic review and meta-analysis
	Epidural	Lusianawati et al. [46]	2023	Tzu Chi Medical Journal	Systematic review and meta-analysis
